# Room temperature lysis incubation improves DNA yield in the Norgen Plant/Fungi DNA Isolation Kit across multiple plant species

**DOI:** 10.1002/aps3.70089

**Published:** 2026-11-08

**Authors:** Shachar Ohali, Jackline Abu‐Nassar, Akiva Shalit‐Kaneh, Emily Draru, Elad Oren

**Affiliations:** ^1^ Newe Ya'ar Research Center, Institute of Plant Sciences, Agricultural Research Organization – Volcani Institute Ramat Yishay 3009500 Israel

**Keywords:** A_260_/A_230_ purity, DNA extraction, genotyping‐by‐sequencing, lysis temperature, Norgen, plant genomics, protocol optimization

## Abstract

**Premise:**

Commercial plant DNA extraction kits typically include a 65°C lysis incubation step, inherited from cetyltrimethylammonium bromide (CTAB)‐based methods. Recent work suggests that cooler lysis temperatures can improve DNA purity in CTAB extractions; whether this also improves yield in CTAB‐based column kits has not been tested.

**Methods and Results:**

We compared room temperature (RT, ~25°C) and 65°C lysis incubation using the Norgen Plant/Fungi DNA Isolation Kit (CTAB‐based lysis buffer) across six plant species in a paired split‐tissue design (*n* = 59 pairs). RT incubation significantly increased Qubit fluorometric DNA yield by 47% overall, with a significant treatment × species interaction. RT‐extracted *Cucurbita pepo* DNA was compatible with genotyping‐by‐sequencing. In a focused generalization test, the modification did not improve yield with the Qiagen DNeasy Plant Mini Kit, whose lysis buffer uses the anionic detergent sodium dodecyl sulphate, indicating that the effect is specific to kit lysis chemistry.

**Conclusions:**

For the Norgen kit, omitting the heating step is a zero‐cost modification that significantly improves DNA yield and the ratio of absorbance at 260 nm and 230 nm (A_260_/A_230_ purity ratio) for most eudicot species tested, with no loss of downstream compatibility. This benefit appears specific to CTAB‐based lysis and should be validated for each kit and species before adoption.

High‐quality genomic DNA is essential for molecular applications in plant biology, including genotyping, marker‐assisted selection, and next‐generation sequencing (Anderson et al., 2018). However, plant DNA extraction is complicated by rigid cell walls and the abundance of secondary metabolites such as polysaccharides and polyphenols, which co‐purify with nucleic acids and inhibit downstream enzymatic reactions (Inglis et al., 2018). Polysaccharide coprecipitation is particularly problematic because it can make the lysate viscous and inhibit downstream enzymatic reactions (Do and Adams, 1991). Commercial spin‐column kits have largely replaced organic solvent–based extraction in many laboratories, offering standardized workflows without hazardous reagents such as chloroform, although they can lead to significant DNA loss on the column (Abdel‐Latif and Osman, 2017). Notably, many of these kits retain a cetyltrimethylammonium bromide (CTAB)‐based lysis step (Doyle and Doyle, 1987), combining CTAB lysis with downstream silica‐membrane purification.

Kit‐based protocols commonly include an initial lysis step at 65°C, which is intended to promote cell lysis and protein denaturation; both the Norgen Plant/Fungi DNA Isolation Kit and the Qiagen DNeasy Plant Mini Kit (Buffer AP1) used here specify a 10‐min incubation at 65°C. This temperature is inherited from CTAB‐based methods, where heating is necessary to maintain CTAB in solution and to facilitate membrane disruption (Doyle and Doyle, 1987; Schenk et al., 2023). Many commercial spin‐column kits retain a CTAB‐based lysis step but introduce chaotropic chemistry downstream, at the DNA‐binding and wash stages rather than at lysis. In the Norgen Plant/Fungi DNA Isolation Kit used here, the lysis buffer (Lysis Buffer L) is CTAB‐based, while guanidinium chloride, a chaotropic salt, is present in the downstream wash solution, according to the manufacturer's Safety Data Sheets. For a CTAB‐based lysis step, the rationale for 65°C incubation is therefore tied in part to CTAB solubility; whether this elevated temperature is necessary, or whether it instead promotes co‐extraction of polysaccharides that become more soluble when heated and reduce the selectivity of binding to silica membranes, has not been systematically evaluated. Carey et al. (2023) experimentally tested lysis temperatures of 50–65°C in conventional CTAB extractions and found that cooler temperatures resulted in significantly higher ratios of absorbance at 260 nm and 230 nm (A_260_/A_230_ purity ratios), although yield differences were not statistically significant. Whether further reducing the incubation temperature—to room temperature—would produce significant yield improvements in column‐based kits that use a CTAB lysis step has not been tested.

During routine extractions using the Norgen Plant/Fungi DNA Isolation Kit, we observed that samples inadvertently processed at room temperature (RT, ~25°C) consistently yielded higher DNA concentrations than those incubated at 65°C. This observation prompted a systematic comparison across six plant species. These species reflect the crops and models that our group and collaborators work with routinely and were therefore readily available; together they span both dicotyledonous and monocotyledonous lineages and include several economically important cucurbit crops, and all are cataloged accessions from publicly accessible germplasm collections. Here, we report that RT lysis incubation significantly improves DNA yield for the majority of species tested, without reducing DNA purity, and demonstrate compatibility with genotyping‐by‐sequencing (GBS).

## METHODS AND RESULTS

### Plant material

Young leaf tissue was collected from six plant species: *Cucurbita pepo* L. (squash; Cucurbitaceae), *Solanum lycopersicum* L. (tomato; Solanaceae), *Citrullus lanatus* (Thunb.) Matsum. & Nakai (watermelon; Cucurbitaceae), *Cucumis melo* L. (melon; Cucurbitaceae), *Arabidopsis thaliana* (L.) Heynh. (Brassicaceae), and *Brachypodium hybridum* Catalán, Joch.Müll., Hasterok & G.Jenkins (Poaceae). Ten biological replicates were collected per species (nine for *Cm. melo* due to one failed sample); five additional *Cb. pepo* accessions were used only in the Qiagen comparison (see below). All plants were grown from seed obtained from publicly accessible, cataloged germplasm collections rather than from wild‐collected material; accession identifiers and source collections for every sample are listed in Appendix S1. Because each sample corresponds to a publicly available accession that uniquely identifies the genetic stock and remains obtainable by other researchers, herbarium vouchers were not deposited; the accession identifiers in Appendix S1 serve as the reproducibility reference for the plant material used. Tissue was flash‐frozen in liquid nitrogen and stored at −80°C until processing.

### DNA extraction and paired design

DNA was extracted using the Norgen Plant/Fungi DNA Isolation Kit (product #26200; Norgen Biotek Corporation, Thorold, Ontario, Canada) following the manufacturer's protocol with two modifications. For each biological replicate, ≤100 mg of frozen tissue was ground to a fine powder in liquid nitrogen using a mortar and pestle. The frozen powder was divided into two equal portions by mass (each weighed on a balance to within ±5 mg) into two pre‐chilled 1.7‐mL microcentrifuge tubes, with the tissue kept frozen on liquid nitrogen throughout. To each tube, 500 µL of Lysis Buffer L and 1 µL of RNase A were added. One tube was incubated at 65°C for 10 min (standard protocol), while the paired tube was incubated at RT (ambient laboratory temperature, ~25°C) for 10 min. All subsequent steps—including addition of 100 µL of Binding Buffer I, 5 min of ice incubation, column filtration, ethanol addition, binding, washing (Solution WN and Wash Solution A), and elution—were performed identically for both conditions according to the manufacturer's instructions. DNA was eluted in 80 µL of Elution Buffer B for both treatments and for all extractions in this study (see Appendix 1 for a step‐by‐step protocol).

### DNA quantification and quality assessment

DNA concentration was measured using a Qubit fluorometer (Thermo Fisher Scientific, Waltham, Massachusetts, USA) with the dsDNA Broad Range assay (primary yield metric) and a NanoDrop spectrophotometer (Thermo Fisher Scientific). Purity was assessed from NanoDrop measurements using two absorbance ratios: A_260_/A_280_, which indexes protein contamination (~1.8–2.0 for pure DNA), and the A_260_/A_230_ ratio, which indexes contamination by polysaccharides, phenolics, and salts (~2.0–2.2 optimal) (Fialova et al., 2020). DNA integrity was visualized by agarose gel electrophoresis. For each sample, 3 µL of extracted DNA was loaded onto a 0.8% (w/v) agarose gel cast in 1× sodium borate buffer (Brody and Kern, 2004), with ethidium bromide added to the molten agarose prior to casting, and run alongside 2 µL of λ DNA/*Hin*dIII size marker (cat. no. SM0101; Thermo Fisher Scientific) at ~100 V for 90 min. Gels were visualized on an ultraviolet (UV) transilluminator‐based gel documentation system (Appendix S2).

### Genotyping‐by‐sequencing validation

To confirm that RT‐extracted DNA is compatible with reduced‐representation sequencing, RT‐extracted DNA from *Cb. pepo* (*n* = 10) was submitted for GBS as a feasibility validation. Libraries were prepared by the Elshire Group Ltd. (Palmerston North, New Zealand) following the Elshire et al. (2011) method: 100 ng of genomic DNA were used, 3.6 ng of total adapters were used, DNA was restricted with *Ape*KI enzyme, and libraries were amplified with 18 PCR cycles. *Ape*KI was selected based on prior GBS studies in *Cucurbita* (Hernandez et al., 2023). Libraries were sequenced on an Illumina platform.

### Statistical analysis

Within‐species treatment effects were assessed using Wilcoxon signed‐rank tests on paired samples. To evaluate the overall treatment effect while accounting for species differences, DNA concentrations were log‐transformed and analyzed using a linear model with treatment, species, and their interaction as fixed effects (JMP Pro; SAS Institute, Cary, North Carolina, USA). Results were confirmed using a Gamma generalized linear mixed model with a log link and sample pair as a random intercept, which produced nearly identical coefficient estimates. Fold change (RT/65°C) and Cohen's *d* (effect size) were calculated per species. Significance was set at α = 0.05.

### Generalization to a second extraction kit

To test whether the effect of RT lysis incubation generalizes beyond the Norgen kit, we performed a focused comparison using the DNeasy Plant Mini Kit (cat. no. 69104; QIAGEN, Hilden, Germany) on 10 samples: five *Cb. pepo* accessions from the same Newe Ya'ar collection (AWM, BBU, BML, CAS, and CIR) and five *Ct. lanatus* samples (accessions C1‐6, C1‐9, C1‐11, C3‐1, and C3‐4), the latter a subset of those used in the main experiment. Tissue powder, prepared and stored at −80°C as in the Norgen experiment (for *Ct. lanatus*, aliquots of the same powder), was divided into paired RT (~25°C) and 65°C portions and extracted following the manufacturer's protocol, with the lysis incubation temperature as the only modification (10 min, as in the main experiment). DNA was eluted in 100 µL of Buffer AE, the kit's standard elution volume. DNA yield was quantified by Qubit (dsDNA Broad Range assay) and NanoDrop as described above. This generalization test was performed several months after the main experiment, using a partially used kit. The RT versus 65°C comparison was assessed within each species using Wilcoxon signed‐rank tests on paired samples; absolute yields were not compared between the two kits, as the experiments differed in elution volume, tissue storage time, kit age, and, for *Cb. pepo*, genotype.

### DNA yield

RT lysis incubation significantly increased DNA yield compared to 65°C across the majority of species tested (Figure 1, Table 1). In the log‐transformed linear model, the overall treatment effect was highly significant (*F*_1,106_ = 25.72, *P* < 0.0001), as was the treatment × species interaction (*F*_5,106_ = 7.80, *P* < 0.0001; *R* ^2^ = 0.81), indicating that the magnitude of improvement varied among species.

**Figure 1:**
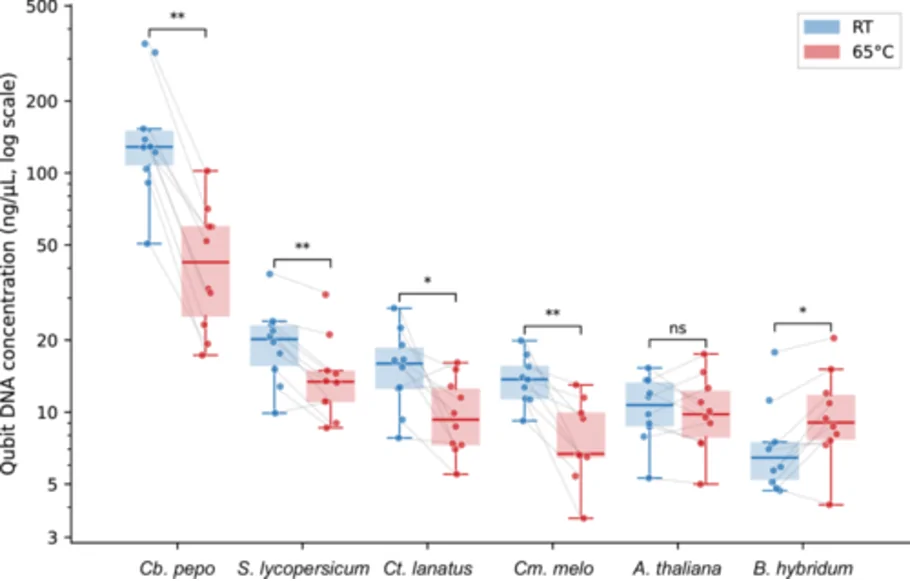
DNA yield (DNA concentration, ng/µL) from room temperature (RT, blue) and 65°C (red) lysis incubation across six plant species (*Cucurbita pepo*, *Solanum lycopersicum*, *Citrullus lanatus*, *Cucumis melo*, *Arabidopsis thaliana*, and *Brachypodium hybridum*). Individual data points from paired samples are connected by gray lines. Boxplots show median, interquartile range, and whiskers; *y*‐axis values are on a log scale. Significance from Wilcoxon signed‐rank tests: *P < 0.05, **P < 0.01, ns = not significant.

**Table 1:** Summary of DNA concentrations (ng/µL) by species and treatment.

		DNA concentration (ng/µL)					
**Species**	***n***	**RT (mean ± SD)**	**65°C (mean ± SD)**	**Fold** a	**Cohen's** ***d***	***P*** **‐value** b	**Significance** c
*Cucurbita pepo*	10	158.2 ± 96.6	46.8 ± 27.1	3.38	1.50	0.002	**
*Solanum lycopersicum*	10	20.3 ± 7.7	14.8 ± 6.7	1.37	1.76	0.004	**
*Citrullus lanatus*	10	16.0 ± 5.9	10.1 ± 3.6	1.58	0.83	0.014	*
*Cucumis melo*	9	13.9 ± 3.3	8.1 ± 3.1	1.72	1.60	0.004	**
*Arabidopsis thaliana*	10	10.7 ± 3.1	10.4 ± 3.7	1.02	0.09	0.922	ns
*Brachypodium hybridum*	10	7.7 ± 4.0	10.4 ± 4.6	0.74	−1.28	0.014	*†

*Note*: RT = room temperature.

^a^ Fold change = RT mean/65°C mean.

^b^ *P*‐values from Wilcoxon signed‐rank tests on paired samples.

^c^ *P < 0.05, **P < 0.01, ns = not significant, †65°C > RT.

The largest yield increase was observed in *Cb. pepo*, where RT incubation produced a 3.4‐fold higher mean DNA concentration (158.2 ± 96.6 vs. 46.8 ± 27.1 ng/µL; Wilcoxon *P* = 0.002). Significant improvements were also found in *Cm. melo* (1.7‐fold; *P* = 0.004), *Ct. lanatus* (1.6‐fold; *P* = 0.014), and *S. lycopersicum* (1.4‐fold; *P* = 0.004). In *A. thaliana*, no significant difference was observed (1.0‐fold; *P* = 0.92). Notably, *B. hybridum* was the only species in which 65°C incubation produced significantly higher yields than RT (0.7‐fold; *P* = 0.014).

Agarose gel electrophoresis corroborated these quantitative differences (Appendix S2). For the eudicot species showing a significant RT advantage, most strikingly *Cb. pepo*, the RT lanes displayed visibly stronger high‐molecular‐weight bands than the paired 65°C lanes, whereas the two treatments produced comparably intense bands for *A. thaliana*, and for *B. hybridum* the 65°C lanes were as strong or stronger than RT, consistent with the Qubit measurements. Across all species, the extracted DNA migrated predominantly as a high‐molecular‐weight band with minimal smearing, indicating that the higher yields obtained at RT were not accompanied by increased degradation.

### DNA purity

A_260_/A_280_ ratios were not significantly affected by incubation temperature (*F*_1,106_ = 2.04, *P* = 0.16), with both conditions producing ratios within the acceptable range of 1.8–2.0 (Figure 2A). In contrast, A_260_/A_230_ ratios were significantly higher under RT conditions (overall increase of +0.35 units; *F*_1,106_ = 20.89, *P* < 0.0001; Figure 2B), with the most pronounced improvement in *Ct. lanatus* (2.1 vs. 1.1) and *Cm. melo* (2.0 vs. 1.3), suggesting reduced co‐purification of polysaccharides or other contaminants at lower lysis temperatures (Fialova et al., 2020).

**Figure 2:**
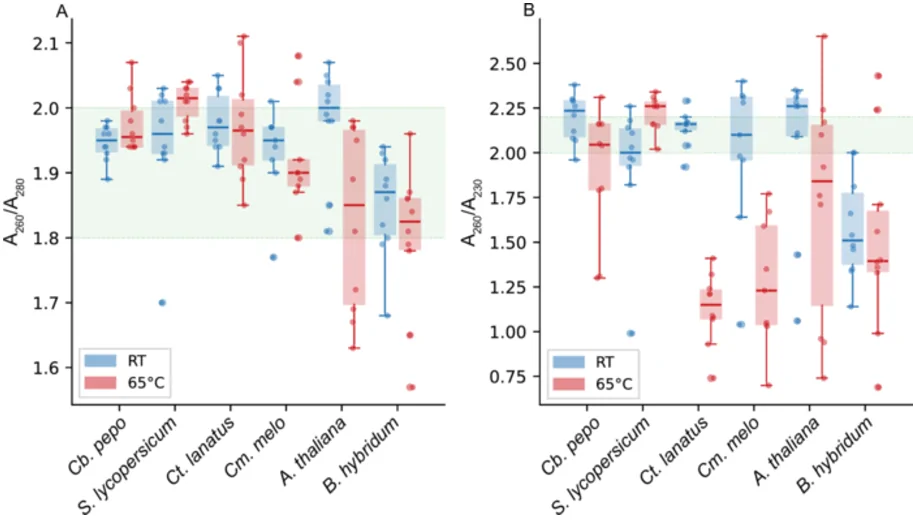
DNA purity ratios from room temperature (RT, blue) and 65°C (red) lysis incubation. (A) A_260_/A_280_ ratios. (B) A_260_/A_230_ ratios. Green shaded regions indicate commonly accepted ideal ranges (1.8–2.0 for A_260_/A_280_; 2.0–2.2 for A_260_/A_230_).

### Qiagen DNeasy results

In contrast to the results obtained with the Norgen kit, RT lysis incubation did not improve DNA yield with the Qiagen DNeasy Plant Mini Kit. For *Cb. pepo* (*n* = 5), DNA concentrations were higher at 65°C than at RT in all five paired samples (mean 9.9 ± 6.4 vs. 4.8 ± 2.3 ng/µL; fold change RT/65°C = 0.48), although this difference was not significant (Wilcoxon signed‐rank *P* = 0.063, the minimum value attainable with *n* = 5). For *Ct. lanatus* (*n* = 5), no significant difference was detected between treatments (5.8 ± 1.3 vs. 4.8 ± 1.4 ng/µL; fold change 0.82; *P* = 0.625). Given the small sample size (*n* = 5), these comparisons are not powered to detect within‐species differences. Per‐sample data are provided in Appendix S3, and agarose gel electrophoresis of the Qiagen‐extracted DNA, consistent with these Qubit measurements, is shown in Appendix S4. These results indicate that the RT lysis advantage observed with the Norgen kit does not extend to the Qiagen kit; the optimal lysis temperature is kit‐dependent.

### GBS validation

All 10 RT‐extracted *Cb. pepo* samples passed library preparation quality control, and sequencing yielded an average of 1.5 million read pairs per sample. After SNP calling, 933,811 sites were genotyped at a mean depth of 5.3 ± 1.7× (range: 2.2–7.6×), with all 10 samples producing usable data, confirming that RT‐extracted DNA is fully compatible with reduced‐representation sequencing workflows.

## CONCLUSIONS

Our results demonstrate that room temperature lysis incubation consistently improves DNA yield for most plant species tested with the Norgen Plant/Fungi DNA Isolation Kit, with no negative effect on DNA purity. These findings extend the observations of Carey et al. (2023), who tested lysis temperatures of 50–65°C in conventional CTAB extractions and found that cooler temperatures significantly improved A_260_/A_230_ purity ratios, although yield differences were not significant. Because the Norgen kit's lysis step is also CTAB‐based, our experiment extends the observation of Carey et al. (2023): reducing the incubation temperature further—to room temperature—produces significant yield improvements when this CTAB‐based lysis is coupled with downstream silica‐column purification. In contrast, the same modification did not improve yield with the Qiagen DNeasy Plant Mini Kit, whose lysis buffer uses an anionic detergent (sodium dodecyl sulphate) rather than CTAB; the optimal lysis temperature therefore depends on the lysis chemistry of the kit, not simply on whether the kit uses a silica column.

The improvement was most pronounced in cucurbit species (*Cb. pepo*, *Ct. lanatus*, *Cm. melo*), which are known to contain high levels of polysaccharides and mucilaginous compounds. We hypothesize that at 65°C, these compounds become more soluble and co‐extract with DNA, interfering with column binding. This interpretation is supported by the significantly lower A_260_/A_230_ ratios under 65°C conditions, as low A_260_/A_230_ values are characteristic of polysaccharide or phenolic contamination (Fialova et al., 2020).

The notable exception was *B. hybridum*, the only monocot in our panel, which showed a modest but significant reversal in favor of 65°C. Monocot cell walls differ structurally from dicots, with higher cellulose content and a distinct hemicellulose composition dominated by arabinoxylans rather than pectins (Biswal et al., 2017). The more rigid cell wall architecture of grasses may require thermal energy for efficient lysis: temperature alone may not capture the full lysis requirement, and the standard 10‐min incubation we used, which was optimized for the manufacturer's 65°C protocol, may be insufficient at room temperature when paired with the more recalcitrant cell walls of grasses. Further testing with additional monocot species and varying lysis durations is needed to determine generalizability and mitigation of reversal.

This modification is particularly relevant when biological material is limited and irreplaceable, for instance, when working with unique F_2_ hybrid individuals from segregating mapping populations, seedlings from accessions with poor germination, or high‐value breeding lines, where the available leaf tissue may be too scarce for a repeat extraction if the first attempt fails. Obtaining 1.5–3.4‐fold more DNA from the same ≤100 mg of tissue can mean the difference between a single PCR attempt and sufficient material for GBS, multiple marker assays, or repeated experiments.

Importantly, RT‐extracted DNA was fully compatible with GBS, confirming that the modification does not compromise DNA quality for next‐generation sequencing. We recommend that users of column‐based plant DNA extraction kits evaluate RT incubation for their specific kit and species of interest, particularly for dicotyledonous plants extracted using CTAB kits.

## AUTHOR CONTRIBUTIONS

This study was conceived and designed by S.O. and E.O., experiments were conducted by S.O., E.D., J.A.N., and A.S.K. The manuscript was written by S.O. and E.O. All authors approved the final version of the manuscript.

## Supporting information


**Appendix S1:** Plant germplasm used in this study. For each sample analyzed in the main experiment, the table lists the sample identifier, species, genotype or accession, source collection, and bibliographic reference for the source collection. *Cucurbita pepo* samples 90–99 correspond to the labels shown in Appendix S2; the *Citrullus lanatus* genotypes correspond to the BC_3_F_4_ introgression lines indicated. *Ct. lanatus* samples are BC_3_F_4_ introgression lines from a *Ct. colocynthis* × *Ct. lanatus* cross; the genome composition is predominantly *Ct. lanatus* (~97%) with introgressed *Ct. colocynthis* segments.

**Appendix S2:** Agarose gel electrophoresis of genomic DNA extracted using the Norgen Plant/Fungi DNA Isolation Kit under room temperature (RT) and 65°C lysis conditions. (A) *Cucurbita pepo* (samples 90–99, paired RT and 65°C lanes; band sizes of the λ/*Hin*dIII ladder are indicated). (B) *Citrullus lanatus*. (C) *Solanum lycopersicum*. (D) *Cucumis melo*. (E) *Arabidopsis thaliana*, shown at two exposure levels because of weaker overall band intensity for this species: (E1) shorter exposure matched to other panels, and (E2) extended exposure with all lanes well above the detection threshold. Within each subpanel, RT and 65°C samples were imaged simultaneously under identical conditions. (F) *Brachypodium hybridum*. For panels B–F, RT samples are in the upper panel and 65°C samples in the lower panel, with 10 samples loaded per condition (9 for *Cm. melo*). λ = lambda DNA size marker.

**Appendix S3:** Paired Qubit DNA concentrations from the Qiagen DNeasy Plant Mini Kit generalization test. (A) *Cucurbita pepo* (*n* = 5); (B) *Citrullus lanatus* (*n* = 5). Red points: 65°C lysis. Blue points: RT lysis. Gray lines connect aliquots from the same paired sample. Sample identifiers correspond to the genotypes listed in Appendix S1. Per‐sample Qubit values (RT vs. 65°C) are tabulated above the figure.

**Appendix S4:** Agarose gel electrophoresis of genomic DNA extracted using the Qiagen DNeasy Plant Mini Kit under room temperature (RT) and 65°C lysis conditions. (A) *Cucurbita pepo* (*n* = 5). (B) *Citrullus lanatus* (*n* = 5). For each species, RT samples are in the upper panel and 65°C samples in the lower panel. λ = lambda DNA size marker. Gel run conditions were identical to those described for Appendix S2.

## Data Availability

All extraction data are provided in the Supporting Information. GBS raw reads will be deposited in the National Center for Biotechnology Information (NCBI) Sequence Read Archive upon publication of a companion study (accession numbers pending).

## Appendix 1 Step‐by‐step protocol: Room temperature lysis modification for the Norgen Plant/Fungi DNA Isolation Kit.

### Materials

- Norgen Plant/Fungi DNA Isolation Kit (Product #26200; Norgen Biotek Corporation, Thorold, Ontario, Canada)
- Fresh or frozen plant leaf tissue (≤100 mg per sample)
- Liquid nitrogen
- Mortar and pestle (pre‐chilled)
- 96–100% ethanol (user‐supplied)
- 70% ethanol (user‐supplied)
- Benchtop microcentrifuge (capable of 20,000 × *g*)
- Vortex mixer
- Analytical balance (for dividing tissue by mass)
- Ice and ice bucket
- Heat block or incubator set to 65°C (for the standard‐protocol comparison)
- Adjustable micropipettes and DNase‐free tips
- DNase‐free 1.7‐mL microcentrifuge tubes

### Procedure

1. Grind ≤100 mg of frozen plant tissue to a fine powder in liquid nitrogen using a pre‐chilled mortar and pestle.
2. Transfer the powder to a DNase‐free 1.7‐mL microcentrifuge tube. Add 500 µL of Lysis Buffer L and 1 µL of RNase A. Vortex for 20 s to mix.
3. MODIFICATION: Incubate at room temperature (~25°C) for 10 min, mixing by inversion 2–3 times during incubation. (Standard protocol: 65°C for 10 min.)
4. Add 100 µL of Binding Buffer I, mix thoroughly, and incubate for 5 min on ice.
5. Assemble a Filter Column (clear O‐ring) with a collection tube. Pipette the lysate into the Filter Column and spin for 2 min at 20,000 × *g*.
6. Transfer the clear supernatant to a new DNase‐free microcentrifuge tube using a pipette.
7. Add an equal volume of 70% ethanol to the supernatant. Vortex to mix.
8. Assemble a Spin Column (gray O‐ring) with a collection tube. Load up to 650 µL of the lysate–ethanol mixture onto the Spin Column and centrifuge for 1 min at 10,000 × *g*. Repeat if necessary for remaining volume.
9. Wash: Apply 500 µL of Solution WN and centrifuge for 1 min at 10,000 × *g*. Discard flowthrough.
10. Wash: Apply 500 µL of Wash Solution A and centrifuge for 1 min at 10,000 × *g*. Discard flowthrough. Repeat once.
11. Dry spin for 2 min at 20,000 × *g* to remove residual ethanol. Discard collection tube.
12. MODIFICATION: Place the column in a fresh 1.7‐mL elution tube. Add 80 µL of Elution Buffer B, incubate 1 min at room temperature, and centrifuge for 1 min at 10,000 × *g*. (Standard protocol: 100 µL of Elution Buffer B.)
13. Store purified DNA at −20°C.

Note: Two steps differ from the manufacturer's standard protocol: Step 3 (lysis temperature) and Step 12 (elution volume reduced to 80 µL to maximize eluate concentration). All other steps are performed identically.
