# Supplementary material for: Room temperature lysis incubation improves DNA yield in the Norgen Plant/Fungi DNA Isolation Kit across multiple plant species
Source: Appl Plant Sci. 2026 Nov 8:e70089. Online ahead of print. doi: 10.1002/aps3.70089 (PMC13745392; doi:10.1002/aps3.70089)

DOI 10.1002/aps3.70089

APPENDIX S2. Agarose gel electrophoresis of genomic DNA extracted using the Norgen Plant/Fungi DNA Isolation Kit under room temperature (RT) and 65°C lysis conditions. (A) *Cucurbita pepo* (samples 90–99, paired RT and 65°C lanes; band sizes of the λ/*Hin*dIII ladder are indicated). (B) *Citrullus lanatus*. (C) *Solanum lycopersicum*. (D) *Cucumis melo*. (E) *Arabidopsis thaliana*, shown at two exposure levels because of weaker overall band intensity for this species: (E1) shorter exposure matched to other panels, and (E2) extended exposure with all lanes well above the detection threshold. Within each subpanel, RT and 65°C samples were imaged simultaneously under identical conditions. (F) *Brachypodium hybridum*. For panels B–F, RT samples are in the upper panel and 65°C samples in the lower panel, with 10 samples loaded per condition (9 for *Cm. melo*). λ = lambda DNA size marker.

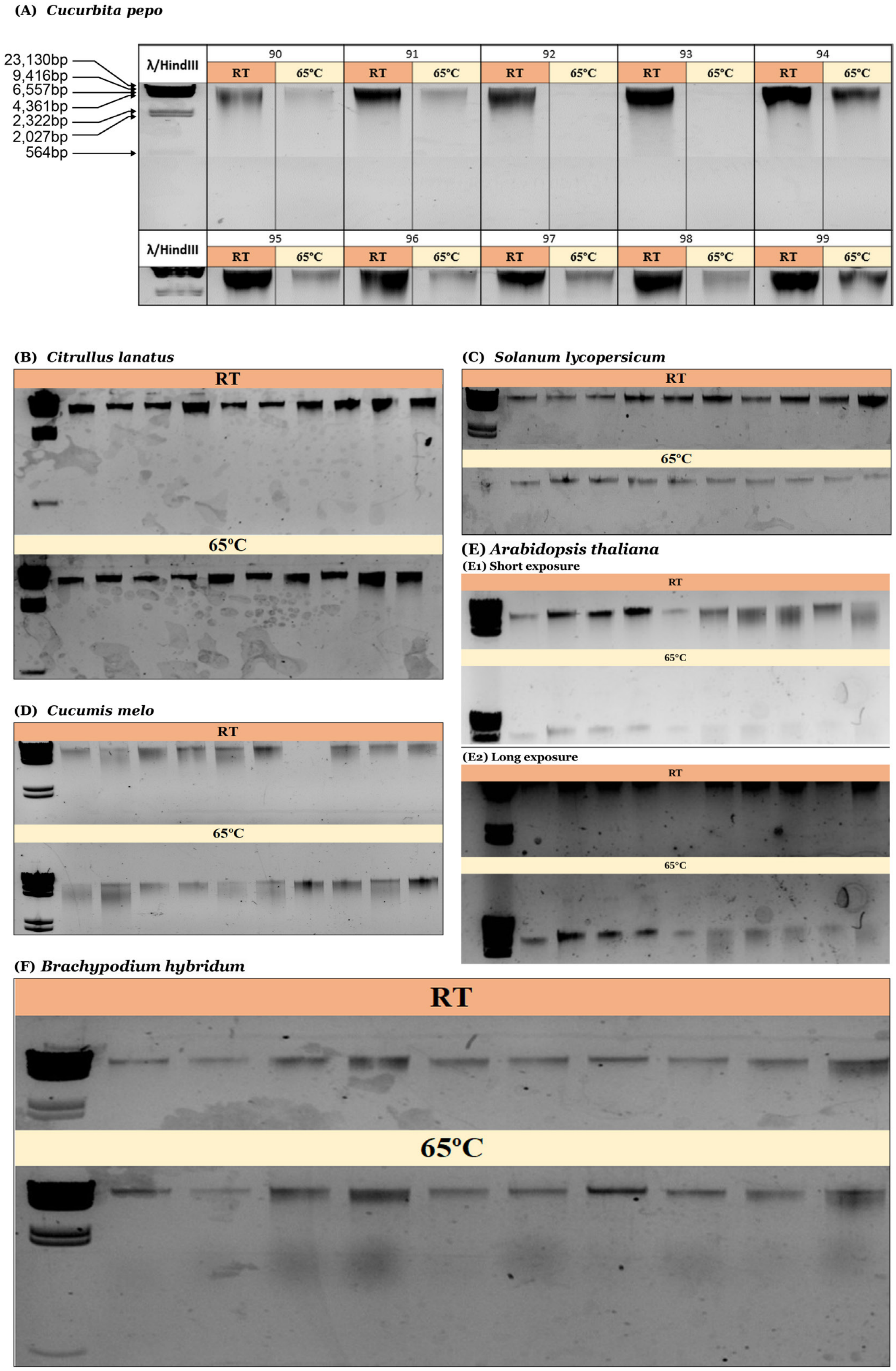

Supplement: Supplementary file 1 [file APS3-9999-e70089-s001.pdf]
