# Supplementary material for: Room temperature lysis incubation improves DNA yield in the Norgen Plant/Fungi DNA Isolation Kit across multiple plant species
Source: Appl Plant Sci. 2026 Nov 8:e70089. Online ahead of print. doi: 10.1002/aps3.70089 (PMC13745392; doi:10.1002/aps3.70089)

DOI 10.1002/aps3.70089

APPENDIX S4. Agarose gel electrophoresis of genomic DNA extracted using the Qiagen DNeasy Plant Mini Kit under room temperature (RT) and 65°C lysis conditions. (A) *Cucurbita pepo* (*n* = 5). (B) *Citrullus lanatus* (*n* = 5). For each species, RT samples are in the upper panel and 65°C samples in the lower panel. λ = lambda DNA size marker. Gel run conditions were identical to those described for Appendix S2.

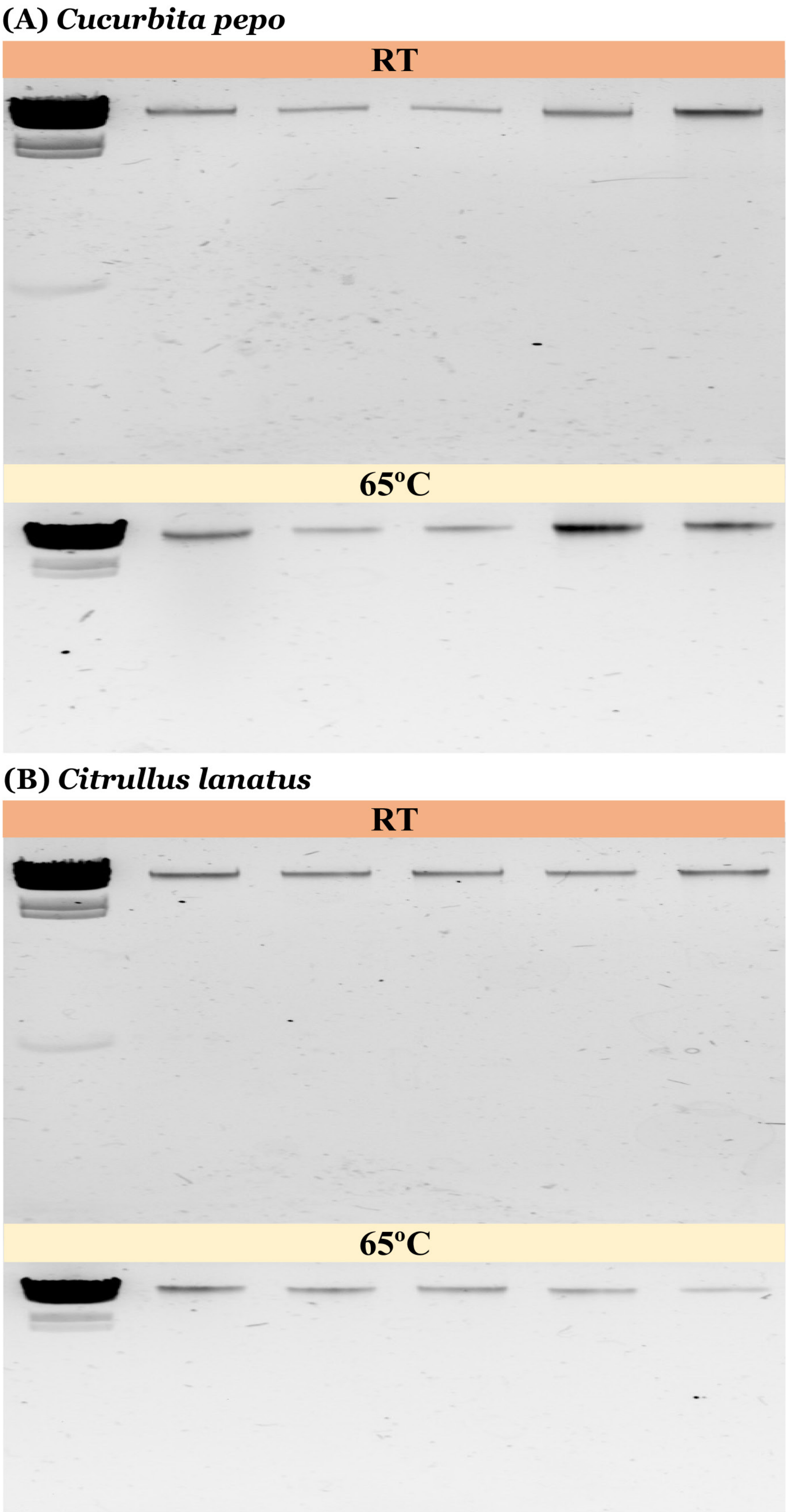

Supplement: Supplementary file 2 [file APS3-9999-e70089-s002.pdf]
