# Supplementary material for: Room temperature lysis incubation improves DNA yield in the Norgen Plant/Fungi DNA Isolation Kit across multiple plant species
Source: Appl Plant Sci. 2026 Nov 8:e70089. Online ahead of print. doi: 10.1002/aps3.70089 (PMC13745392; doi:10.1002/aps3.70089)

DOI 10.1002/aps3.70089

APPENDIX S3. Paired Qubit DNA concentrations from the Qiagen DNeasy Plant Mini Kit generalization test. (A) *Cucurbita pepo* (*n* = 5); (B) *Citrullus lanatus* (*n* = 5). Red points: 65°C lysis. Blue points: RT lysis. Gray lines connect aliquots from the same paired sample. Sample identifiers correspond to the genotypes listed in Appendix S1. Per-sample Qubit values (RT vs. 65°C) are tabulated above the figure.

Sample ID	Species	Qubit DNA concentration (ng/µL)	
		65°C	RT
AWM	*Cb. pepo*	9.95	4.91
CIR	*Cb. pepo*	9.55	7.73
BML	*Cb. pepo*	4.76	2.51
CAS	*Cb. pepo*	20.40	6.30
BBU	*Cb. pepo*	4.95	2.46
C1-6	*Ct. lanatus*	6.53	5.04
C1-9	*Ct. lanatus*	5.94	3.76
C1-11	*Ct. lanatus*	7.14	5.58
C3-1	*Ct. lanatus*	5.91	3.00
C3-4	*Ct. lanatus*	3.66	6.63

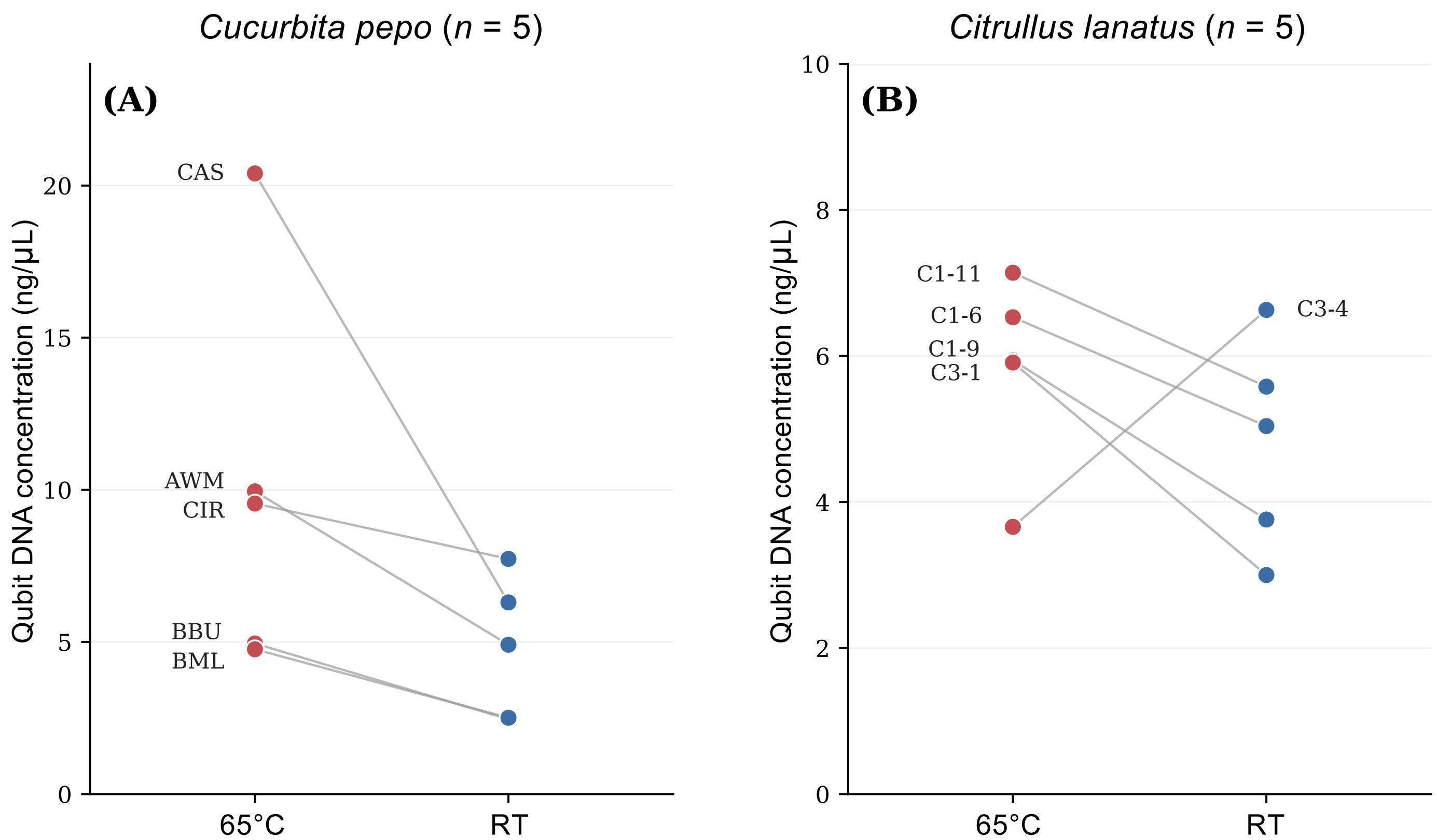

Supplement: Supplementary file 3 [file APS3-9999-e70089-s004.pdf]
